# Adjuvanted recombinant hemagglutinin H7 vaccine to highly pathogenic influenza A(H7N9) elicits high and sustained antibody responses in healthy adults

**DOI:** 10.1038/s41541-021-00287-7

**Published:** 2021-03-19

**Authors:** Christine M. Oshansky, James King, Di Lu, James Zhou, Corrina Pavetto, Gary Horwith, Karen Biscardi, Bai Nguyen, John J. Treanor, Li-Mei Chen, Brett Jepson, BPI17002 Study Coordination Team, Chad Colfer, Penny Hylton, James Little, Michael O’Hara, Silvija Tresnjak-Smith, Robert Walker, Bai Yeh, Rick A. Bright, Robert A. Johnson, Vittoria Cioce, Ruben O. Donis

**Affiliations:** 1 Biomedical Advanced Research and Development Authority (BARDA), Office of the Assistant Secretary for Preparedness and Response (ASPR), Department of Health and Human Services (HHS), Washington, DC USA; 2 Rho, Inc., Durham, NC USA

**Keywords:** Infectious diseases, Influenza virus, Influenza virus, Adjuvants, Protein vaccines

## Abstract

An unprecedented number of human infections with avian influenza A(H7N9) in the fifth epidemic wave during the winter of 2016–2017 in China and their antigenic divergence from the viruses that emerged in 2013 prompted development of updated vaccines for pandemic preparedness. We report on the findings of a clinical study in healthy adults designed to evaluate the safety and immunogenicity of three dose levels of recombinant influenza vaccine derived from highly pathogenic A/Guangdong/17SF003/2016 (H7N9) virus adjuvanted with AS03 or MF59 oil-in water emulsions. Most of the six study groups meet the FDA CBER-specified vaccine licensure criterion of 70% seroprotection rate (SPR) for hemagglutination inhibition antibodies to the homologous virus. A substantial proportion of subjects show high cross-reactivity to antigenically distinct heterologous A(H7N9) viruses from the first epidemic wave of 2013. These results provide critical information to develop a pandemic response strategy and support regulatory requirements for vaccination under Emergency Use Authorization.

## Introduction

Zoonotic infections with a novel Asian lineage avian-origin influenza A(H7N9) virus were first reported in China in March 2013 and caused severe, often fatal, lower respiratory tract disease in humans^1,2^. Since then, influenza A(H7N9) virus was found to be circulating in poultry in China, and until 2017, human epidemics have been reported annually between fall and early spring^3^. During the fifth epidemic in the winter of 2016–2017, an unprecedented number of human influenza A(H7N9) cases were identified. Genetic and antigenic analyses indicated that more than 90% of the circulating H7N9 viruses belonged to a new group designated Yangtze River Delta lineage, antigenically distinct from the previously dominant Pearl River Delta lineage^4^. The new group had reduced cross-reactivity with antibodies raised to existing candidate vaccine viruses (CVVs) made in 2013, prompting the World Health Organization (WHO) to update the pandemic influenza vaccine recommendations^5^. Although the influenza A(H7N9) viruses that emerged in 2013 were characterized by having low pathogenicity in chickens (low pathogenic avian influenza or LPAI), some of the Yangtze River Delta lineage viruses emerging in late 2016 were highly pathogenic for poultry (highly pathogenic avian influenza; HPAI) and caused several human infections^3,6^. Furthermore, mutations in some A(H7N9) viruses detected in humans resulted in reduced susceptibility to influenza antiviral drugs, increasing the potential public health impact of these viruses^7^. Consequently, global health authorities convened by the WHO recommended development of CVVs based on a LPAI A/Hong Kong/125/2017-like virus and a HPAI A/Guangdong/17SF003/2016-like virus^5^, hereafter referred to as A/HK/2017 and A/GD/2016, respectively.

The United States (US) Department of Health and Human Services (HHS) continuously monitors pandemic risk and prepares to respond to the threat of novel influenza virus outbreaks in the US. To this end, the Biomedical Advanced Research and Development Authority (BARDA), within the Office of the Assistant Secretary for Preparedness and Response (ASPR), has established and maintains the National Pre-Pandemic Influenza Vaccine Stockpile (NPIVS) comprising adjuvants and pre-pandemic bulk antigens from avian influenza viruses determined to pose a significant risk for a pandemic. In addition, to shorten timelines to make influenza vaccines available to immunize the US population, HHS has supported expansion of domestic vaccine manufacturing capacity, including the use of adjuvanted vaccines, and the licensure of pre-pandemic and seasonal egg-based, cell-based, and recombinant influenza vaccines. While egg- and cell-based novel influenza vaccine antigens have been clinically evaluated with adjuvants AS03 and MF59 to support their deployment for pandemic mitigation under Emergency Use Authorization by the FDA^8–17^, safety and dose-sparing evidence of these adjuvants with recombinant H7 hemagglutinin (HA) antigens is lacking. The present study closed this gap by evaluating the safety and immunogenicity of adjuvanted influenza H7 vaccine derived from A/GD/2016 utilizing recombinant protein technology that can be used to respond quickly to a pandemic influenza virus. By formulating the recombinant protein vaccine with adjuvants from the NPIVS, the results from this study provide critical insights for the US Government to develop a response strategy for a pandemic emergency.

## Results

### Study population

**Fig. 1:**
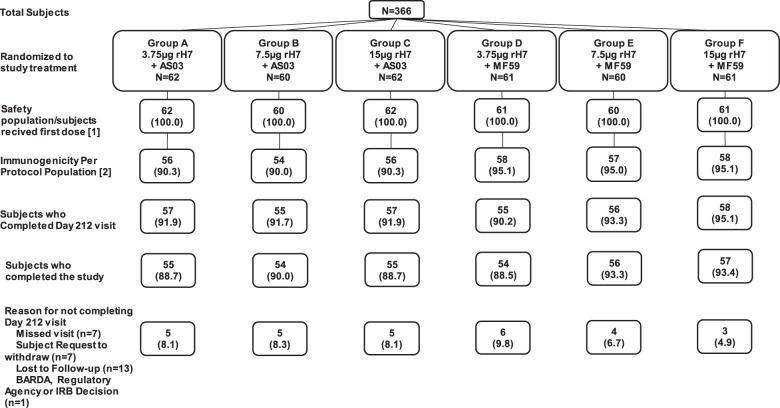
Subject enrollment and randomization are shown by treatment group. The Safety Population consisted of all subjects who were randomly assigned and received at least one dose of vaccine. The immunogenicity per protocol population (IPPP) included subjects who received two full doses of randomized vaccine, had valid hemagglutination inhibition (HAI) results at the day 50 visit (primary immunogenicity endpoint), and had no major protocol deviations that might impact the assessment of immunogenicity.

A total of 366 subjects were enrolled and randomly assigned to each treatment group (Fig. 1 and Table 1). Subjects received two doses of monovalent recombinant influenza H7 vaccine derived from A/GD/2016 at three antigen dose levels (3.75, 7.5, and 15 µg) adjuvanted with AS03 or MF59 administered 28 days apart. The mean subject age was 35.9 years (range: 18–49 years), 55.2% female (range: 48.4–61.7%), 79.5% white (range: 70.0–86.9%), and 16.4% Black or African American (range: 8.2–28.3%). Treatment compliance was high for both vaccine administrations. All subjects who received at least one vaccine dose were considered part of the safety population, while 339 subjects who received both vaccine doses and completed the primary immunogenicity endpoint at day 50, comprised the immunogenicity per protocol population.

**Table 1:** Safety population demographics.

	Study group^a^							
	AS03				MF59			
	3.75 µg HA	7.5 µg HA	15 µg HA	Adjuvant total	3.75 µg HA	7.5 µg HA	15 µg HA	Adjuvant total
	*n* = 62	*n* = 60	*n* = 62	*n* = 184	*n* = 61	*n* = 60	*n* = 61	*n* = 182
Age (years)								
* n*	62	60	62	184	61	60	61	182
Mean (SD)	35.2 (7.90)	34.8 (9.09)	36.4 (9.31)	35.5 (8.76)	35.5 (9.57)	37.6 (7.74)	35.7 (8.18)	36.3 (8.54)
Median	34.6	34.2	36.5	35.4	36.7	38.7	36.2	37.2
Min, Max	19, 49	20, 49	18,49	18, 49	18, 49	21, 49	20, 49	18, 49
Sex, *n* (%)								
Male	30 (48.4%)	29 (48.3%)	32 (51.6%)	91 (49.5%)	24 (39.3%)	23 (38.3%)	26 (42.6%)	73 (40.1%)
Female	32 (51.6%)	31 (51.7%)	30 (48.4%)	93 (50.5%)	37 (60.7%)	37 (61.7%)	35 (57.4%)	109 (59.9%)
Race^b^, *n* (%)								
White	50 (80.6%)	42 (70.0%)	52 (83.9%)	144 (78.3%)	51 (83.6%)	43 (71.7%)	53 (86.9%)	147 (80.8%)
Black or African American	8 (12.9%)	17 (28.3%)	8 (12.9%)	33 (17.9%)	9 (14.8%)	13 (21.7%)	5 (8.2%)	27 (14.8%)
Asian	1 (1.6%)	1 (1.7%)	0 (0.0%)	2 (1.1%)	0 (0.0%)	1 (1.7%)	1 (1.6%)	2 (1.1%)
American Indian or Alaska Native	0 (0.0%)	0 (0.0%)	2 (3.2%)	2 (1.1%)	0 (0.0%)	2 (3.3%)	0 (0.0%)	2 (1.1%)
Native Hawaiian or Other Pacific Islander	0 (0.0%)	0 (0.0%)	0 (0.0%)	0 (0.0%)	0 (0.0%)	0 (0.0%)	0 (0.0%)	0 (0.0%)
More than one race	3 (4.8%)	0 (0.0%)	0 (0.0%)	3 (1.6%)	1 (1.6%)	1 (1.7%)	2 (3.3%)	4 (2.2%)
Ethnicity, *n* (%)								
Hispanic or Latino	2 (3.2%)	6 (10.0%)	7 (11.3%)	15 (8.2%)	1 (1.6%)	2 (3.3%)	4 (6.6%)	7 (3.8%)
Not Hispanic or Latino	60 (96.8%)	54 (90.0%)	55 (88.7%)	169 (91.8%)	60 (98.4%)	58 (96.7%)	57 (93.4%)	175 (96.2%)
Body mass index (kg/m^2^)^c^								
* n*	62	60	62	184	61	60	61	182
Mean (SD)	26.43 (4.332)	27.22 (3.988)	27.65 (4.601)	27.1 (4.324)	26.59 (4.635)	26.93 (4.593)	27.86 (4.206)	27.12 (4.489)
Median	25.70	27.45	27.90	26.90	26.10	27.50	28.30	27.50
Range (Min, Max)	(18.4, 34.8)	(19.8, 34.1)	(18.8, 34.9)	(18.4, 34.9)	(17.5, 34.8)	(16.0, 34.7)	(17.3, 34.6)	(16.0, 34.8)

Abbreviations: min, minimum; max, maximum; *SD*, standard deviation.

^a^Study groups were defined by the first and second vaccinations received by subjects.

^b^Subjects with more than one race category recorded on the case report form appear in the multiracial category.

^c^Body mass index = weight (kg)/[height (m)²].

### Safety and tolerability

**Fig. 2:**
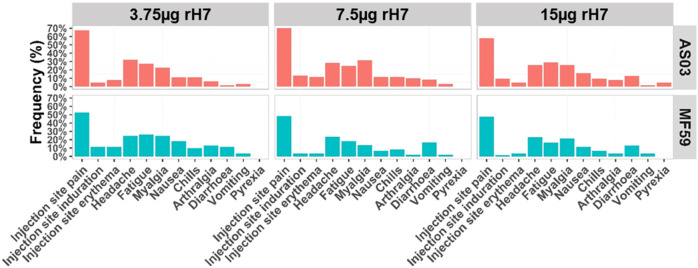
Frequency of adverse events by vaccine received. Solicited local reactions at the injection site included pain, induration, and erythema. Solicited systemic reactions included headache, fatigue, myalgia, nausea, chills, arthralgia, diarrhoea, vomiting, and pyrexia.

The adjuvanted recombinant H7 vaccines were well-tolerated regardless of antigen dose (Fig. 2 and Table 2), and there was no evidence of increased frequencies of subjects experiencing adverse events (AEs) with increasing dose. In general, the frequencies and distribution of local and systemic AEs were similar between adjuvants. Most solicited local and systemic AEs were considered mild to moderate severity. The most common solicited AE for local reactogenicity was injection site pain (Fig. 2). There were no AEs of special interest or potentially immune-mediated medical conditions (PIMMCs) reported. There were no serious AEs (SAEs) related to vaccination as defined by 21 CFR 312.32(a), and there were no AEs or SAEs that led to study withdrawal. Finally, no significant differences were observed in changes from baseline for clinical laboratory results or vital signs for any of the study groups (data not shown).

**Table 2:** Summary of all adverse events within the safety population.

	AS03						MF59					
	3.75 µg HA		7.5 µg HA		15 µg HA		3.75 µg HA		7.5 µg HA		15 µg HA	
	Dose 1^a^	Dose 2^b^	Dose 1^a^	Dose 2^b^	Dose 1^a^	Dose 2^b^	Dose 1^a^	Dose 2^b^	Dose 1^a^	Dose 2^b^	Dose 1^a^	Dose 2^b^
	*N* = 62	*N* = 60	*N* = 60	*N* = 58	*N* = 62	*N* = 60	*N* = 61	*N* = 60	*N* = 60	*N* = 59	*N* = 61	*N* = 60
	*n* (%)^c^	*n* (%)^c^	*n* (%)^c^	*n* (%)^c^	*n* (%)^c^	*n* (%)^c^	*n* (%)^c^	*n* (%)^c^	*n* (%)^c^	*n* (%)^c^	*n* (%)^c^	*n* (%)^c^
Any adverse event	48	39	44	37	40	34	45	29	40	29	37	29
	(77.4)	(65.0)	(73.3)	(63.8)	(64.5)	(56.7)	(73.8)	(48.3)	(66.7)	(49.2)	(60.7)	(48.3)
Any solicited local reactogenicity symptom	39	31	36	34	33	29	33	16	27	16	25	20
	(62.9)	(51.7)	(60.0)	(58.6)	(53.2)	(48.3)	(54.1)	(26.7)	(45.0)	(27.1)	(41.0)	(33.3)
Mild to moderate^d^	39	31	35	33	32	28	33	16	27	15	25	20
	(62.9)	(51.7)	(58.3)	(56.9)	(51.6)	(46.7)	(54.1)	(26.7)	(45.0)	(25.4)	(41.0)	(33.3)
Severe^d^	0	0	1	1	1	1	0	0	0	1	0	0
	(0.0)	(0.0)	(1.7)	(1.7)	(1.6)	(1.7)	(0.0)	(0.0)	(0.0)	(1.7)	(0.0)	(0.0)
Any solicited systemic reactogenicity symptom	20	15	22	17	18	19	22	13	20	12	18	14
	(32.3)	(25.0)	(36.7)	(29.3)	(29.0)	(31.7)	(36.1)	(21.7)	(33.3)	(20.3)	(29.5)	(23.3)
Mild to moderate^d^	18	15	22	17	17	18	19	13	20	12	18	14
	(29.0)	(25.0)	(36.7)	(29.3)	(27.4)	(30.0)	(31.1)	(21.7)	(33.3)	(20.3)	(29.5)	(23.3)
Severe^d^	2	0	0	0	1	1	3	0	0	0	0	0
	(3.2)	(0.0)	(0.0)	(0.0)	(1.6)	(1.7)	(4.9)	(0.0)	(0.0)	(0.0)	(0.0)	(0.0)
Any unsolicited adverse event	17	12	17	10	16	10	15	16	18	9	13	11
	(27.4)	(20.0)	(28.3)	(17.2)	(25.8)	(16.7)	(24.6)	(26.7)	(30.0)	(15.3)	(21.3)	(18.3)
Any severe adverse event	6	3	5	4	4	2	7	2	6	6	2	3
	(9.7)	(5.0)	(8.3)	(6.9)	(6.5)	(3.3)	(11.5)	(3.3)	(10.0)	(10.2)	(3.3)	(5.0)
Any serious adverse event	0	0	0	0	0	0	0	1	0	0	0	0
	(0.0)	(0.0)	(0.0)	(0.0)	(0.0)	(0.0)	(0.0)	(1.7)	(0.0)	(0.0)	(0.0)	(0.0)
Any adverse event related to vaccination	43	34	41	34	35	31	41	21	36	19	34	26
	(69.4)	(56.7)	(68.3)	(58.6)	(56.5)	(51.7)	(67.2)	(35.0)	(60.0)	(32.2)	(55.7)	(43.3)
Any adverse event leading to early study withdrawal	0	0	0	0	0	0	0	0	0	0	0	0
	(0.0)	(0.0)	(0.0)	(0.0)	(0.0)	(0.0)	(0.0)	(0.0)	(0.0)	(0.0)	(0.0)	(0.0)

^a^Adverse events beginning after the 1st dose on day 1, and before the 2nd dose on day 29 (or day 50 if the 2nd dose was not given) are included.

^b^Adverse events beginning after the 2nd dose on day 29 and before day 53 (which is the upper bound of the visit window for Visit 7/day 50) are included.

^c^Percentage of subjects among treatment group.

^d^Subjects reporting more than one adverse event are only counted once at the maximum severity. Mild = Grade 1; Moderate = Grade 2; Severe = Grades 3, 4, and 5.

### Immunogenicity

The purpose of this study was to inform the US Department of Health and Human Services (HHS) Pandemic Influenza Plan preparedness and response strategy. Therefore, any comparisons between different treatment groups were outside the scope and intent of this study and not performed. The primary immunogenicity objective of the study was to determine seroprotection rates (SPRs) in healthy adults following two doses of adjuvanted recombinant H7 vaccine based on serum hemagglutination inhibition (HAI) antibody titers on day 50, defined as an HAI antibody titer ≥1:40 against two representative A(H7N9) influenza viruses which emerged during the fifth epidemic and co-circulated in China: the HPAI A/GD/2016 virus used in the vaccine and LPAI A/HK/2017 virus.

**Fig. 3:**
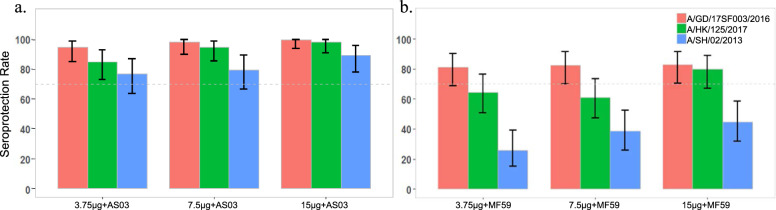
Seroprotection rates (SPRs) to homologous and heterologous H7N9 influenza viruses. Recombinant H7 vaccination adjuvanted with AS03 (**a**) or MF59 (**b**) at 3.75, 7.5, and 15â€‰µg HA doses induced seroprotection on day 50 to homologous A/Guangdong/17SF003/2016 (H7N9) and heterologous A/Hong Kong/125/2017 (H7N9) fifth epidemic viruses and heterologous A/Shanghai/02/2013 (H7N9) first epidemic virus. Data are shown as the proportion of subjects achieving a serum hemagglutination inhibition (HAI) antibody titer of at least 1:40 against the antigen as measured by HAI antibody titers (SPR, seroprotection rate, and 95% CI; vertical bars).

In subjects receiving recombinant H7 + AS03, seroprotection was achieved against the homologous vaccine virus (A/GD/2016) in 94.6%, 98.1%, and 100% of subjects receiving a 3.75, 7.5, and 15 µg dose, respectively, with an overall SPR of 97.6% (95% CI: 93.9–99.3%) (Fig. 3a). recombinant H7 + MF59 elicited seroprotection against the vaccine virus in 81.0%, 82.5%, or 82.8% of subjects receiving a 3.75, 7.5, or 15 µg dose, respectively, with an overall SPR of 82.1% (95% CI: 75.5–87.5%) (Fig. 3b). The lower bounds of the 95% CI around the SPR were ≥70% (the FDA SPR criterion for licensure of pandemic influenza vaccines^18^) in all groups except the 3.75 µg + MF59 group, in which the lower bound of the 95% CI around the SPR was 68.6%. Seroprotection was also observed against the antigenically related A/HK/2017 (H7N9) virus in 85.7%, 96.3%, or 98.2% of subjects receiving recombinant H7 + AS03 at a 3.75, 7.5, or 15 µg dose, respectively, with an overall SPR of 93.4% (95% CI: 88.5–96.6%) and in 63.8%, 61.4%, or 81.0% of subjects receiving a 3.75, 7.5, or 15 µg dose recombinant H7 + MF59, respectively, with an overall SPR of 68.8% (95% CI: 61.3–75.6%) (Fig. 3a, b; green bars).

Notably, adjuvanted recombinant H7 elicited strong cross-reactive antibody responses measured by HAI to the antigenically distant A/Shanghai/02/2013 (H7N9) heterologous virus, hereafter referred to as A/SH/2013, from the first epidemic wave. Recombinant H7 + AS03 at a 3.75, 7.5, or 15 µg dose induced seroprotection at day 50 in 76.8%, 79.6%, or 89.3% of subjects, respectively (overall SPR 81.9%, 95% CI: 75.2–87.5%) and in 25.9%, 38.6%, or 44.8% of subjects receiving recombinant H7 + MF59 at a 3.75, 7.5, or 15 µg dose (overall SPR 36.4%, 95% CI: 29.2–44.1%) (Fig. 3a, b; blue bars).

**Fig. 4:**
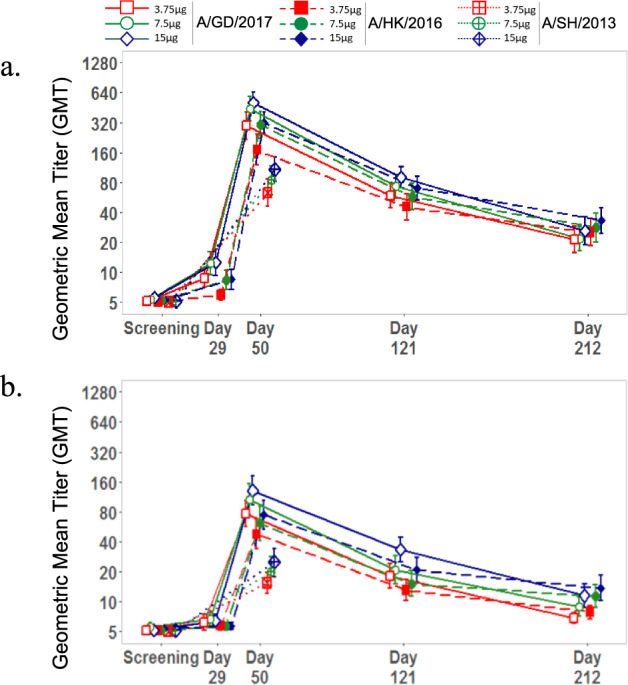
Adjuvanted recombinant H7 at 3.75, 7.5, and 15 µg doses elicits robust antibody responses in all study groups. Hemagglutination inhibition (HAI) antibody responses to homologous A/Guangdong/17SF003/2016 (H7N9) and heterologous A/Hong Kong/125/2017 (H7N9) fifth epidemic viruses as well as heterologous A/Shanghai/02/2013 (H7N9) first epidemic virus (days 1 and 50 only) following AS03-adjuvanted recombinant H7 (a) and MF59-adjuvanted recombinant H7 (b) vaccination. Data are shown as geometric mean titer (GMT); 95% confidence interval (CI; vertical bars).

**Fig. 5:**
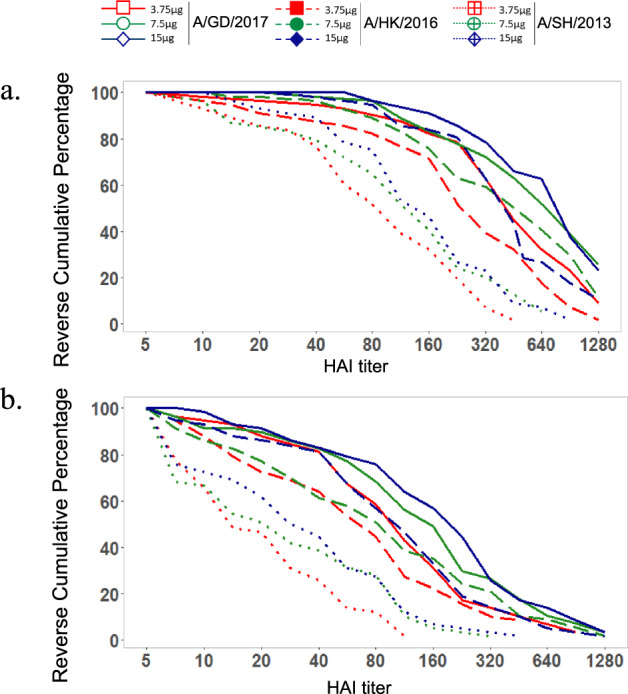
Reverse cumulative percentages for serum HAI antibody responses generated by adjuvanted recombinant H7 vaccination. AS03-adjuvanted recombinant H7 (**a**) and MF59-adjuvanted recombinant H7 (**b**) to homologous A/Guangdong/17SF003/2016 (H7N9) and heterologous A/Hong Kong/125/2017 (H7N9) fifth epidemic viruses as well as heterologous A/Shanghai/02/2013 (H7N9) first epidemic virus (days 1 and 50 only) vaccination.

**Fig. 6:**
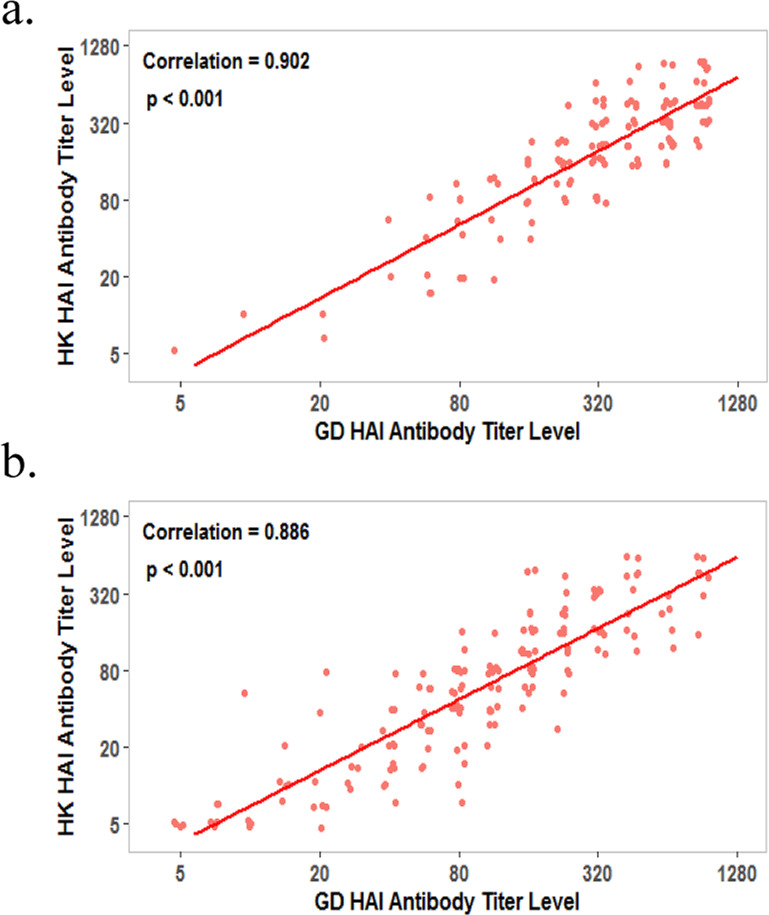
Serum hemagglutination inhibition antibody responses to each of the two fifth epidemic H7N9 strains tested were highly correlated. HAI antibody titers generated by AS03-adjuvanted recombinant H7 (**a**) and MF59-adjuvanted recombinant H7 (**b**) against HPAI and LPAI H7N9 viruses from the fifth epidemic.

As expected, HAI and microneutralization (MN) geometric mean titers (GMTs) against both the A/GD/2016 and A/HK/2017 strains peaked by day 50 but still remained above baseline 6 months later (day 212) for each study group, regardless of antigen dose (Fig. 4a, b and Supplementary Fig. S1). A modest response to antigen dose escalation was observed, with greater antibody titers corresponding to higher antigen doses. Reverse cumulative percentages for serum HAI titers achieved at day 50 following adjuvanted recombinant H7 vaccination show that antigen dose likely enhances HAI and neutralizing antibody responses within the confines of the quantification limits for the respective assays (Fig. 5a, b; Table 3). Similar trends were observed with MN titers, and MN antibody GMTs correlated closely with HAI antibody GMTs (Supplementary Fig. S1; data not shown). The HAI titers against each of the two fifth epidemic H7N9 strains tested were highly correlated (*r* = 0.88 or 0.90; *p* < 0.001) (Fig. 6a, b).

**Table 3:** Serology assay sensitivity: lower and upper antibody titers that could be determined with acceptable precision and relative accuracy.

Influenza virus strain	Assay	LLOQ	ULOQ	LOD
A/Guangdong/17SF003/2016xPR8 (H7N9)	HAI	22.45	1280	<10
	MN	6.60	685.94	<10
A/Hong Kong/125/2017xPR8 (H7N9)	HAI	15.87	958.92	<10
	MN	10	1076.35	<10
A/Shanghai/02/2013xPR8 (H7N9)	HAI	20	nd	14.29
	MN	10	nd	<10

*LLOQ* lower limit of quantitation, *ULOQ* upper limit of quantitation, *LOD* limit of detection, *nd* not done.

## Discussion

Several policy documents from agencies of the US government establish a preparedness goal of maintaining enough pre-pandemic antigen and adjuvants to rapidly formulate vaccine from the NPIVS to vaccinate 26 million persons as well as to expand the domestic vaccine manufacturing capacity to produce enough vaccine for the entire US population within 6 months of a pandemic declaration^19–21^. More recently, the HHS Pandemic Influenza Plan was updated to establish delivery of first doses of pandemic vaccine within 12 weeks of a pandemic declaration^22^. Because recombinant influenza vaccines can proceed on virus sequence alone and do not require prior biosafety assessment and WHO distribution of a candidate vaccine virus to begin manufacturing^23,24^, the platform is expected to accelerate the time to release of product and thus be critical for a timely and effective pandemic response.

Following the first A(H7N9) epidemic in early 2013, clinical studies were quickly launched and showed that two doses of adjuvanted, inactivated egg- or cell-based A/SH/2013 (H7N9) vaccines induced HAI and neutralizing antibody titers to homologous antigen^12,14,17^. In particular, subjects achieved SPRs of 91%, 81%, and 84% and HAI antibody GMTs of 107.1, 80.9, and 103.4 following two doses of AS03-adjuvanted A/SH/2013 (H7N9) vaccine at doses of 3.75, 7.5, and 15 µg of HA, respectively^12^. Two doses of MF59-adjuvanted A/SH/2013 vaccine elicited seroprotection in 59%, 58%, and 47% of subjects and HAI antibody GMTs of 33.0, 33.8, and 25.3 at doses of 3.75, 7.5, and 15µg of HA, respectively^14^. Similar responses were observed in subjects administered two doses of MF59-adjuvanted egg- or cell-based A/SH/2013 (H7N9) vaccine^12,17^.

Since A(H7N9) influenza emerged in February 2013, the WHO has confirmed 1568 cases of avian influenza A(H7N9) as of June 18, 2020^25^. The A(H7N9) epidemic in 2016–2017 in China was unprecedented, with a total of 758 cases and 288 deaths reported^26^. This, combined with the acquisition of HPAI properties, identification of travel-related cases outside China, the potential loss of protection by the A/SH/2013 (H7N9) vaccine in the US NPIVS, and a high frequency of viruses with reduced sensitivity to neuraminidase inhibitors (~10%) provided a strong driving force to strengthen the US National preparedness position. Thus, BARDA initiated development and production of new H7N9 influenza vaccine antigens in different manufacturing platforms (egg-based, cell-based, and recombinant) taking into consideration the vaccine composition recommendations of WHO. The HPAI A/GD/2016 H7N9 virus was selected to produce vaccine antigen using the Flublok manufacturing process licensed in the US, by Protein Sciences Corporation.

Early antigenic analyses of fifth wave A(H7N9) influenza viruses published by the WHO indicated that the HAI titers of ferret antisera to the A/SH/2013 were substantially lower than the homologous titers^5^. In contrast, ferret antisera to the newly designated fifth wave CVVs, especially to the HPAI A/GD/2016 virus, were broadly reactive with all contemporary H7N9 viruses, including LPAI and HPAI viruses collected in 2016–2017 from humans and birds. These studies in ferrets led us to expect that recombinant H7 vaccine produced from the HPAI virus sequence would elicit highly cross-reactive antibody responses to a majority of the fifth epidemic emergent viruses in subjects who receive two doses with adjuvant. Furthermore, vaccine formulation with adjuvant may be able to overcome significant antigenic distance as the viruses evolve in subsequent years. In the absence of such viruses, antigenically distant ancestor viruses can be used for this purpose in assays; i.e., viruses that circulated during the first H7N9 epidemics in 2013. Indeed, strong cross-reactive antibody responses were observed to the antigenically distant A/Shanghai/2/2013 (H7N9) first epidemic strain suggesting that antibodies elicited by this adjuvanted vaccine may provide adequate protection for viruses evolving antigenically in the coming years. In agreement with these findings, three of six groups achieved seroprotection against the heterologous but antigenically related A/Hong Kong/125/2017 (H7N9) from the fifth epidemic wave. Furthermore, SPRs persisted near or above 70% at 3 months and remained above baseline at 6 months post-vaccination. The cross-reactive immune responses observed here and in other studies^27–30^ underscore the importance of continual testing of vaccines for candidate influenza viruses with high risk of pandemic potential. Sera obtained from vaccines that administered the A/SH/2013 (H7N9) antigen did not highly cross-react to fifth epidemic H7N9 viruses (Levine et al., 2017, personal communication), however in this study we show that subjects receiving Recombinant H7 had relatively high cross-reactive antibody responses to the only distantly antigenically related A/SH/2013 (H7N9). This one-way loss of reactivity is not completely understood, but the purity of the recombinant vaccine antigen may have a role. The National Institutes of Health (NIH), National Institute of Allergy and Infectious Diseases (NIAID) has several vaccine studies in progress using egg-based H7N9 vaccine derived from the LPAI A/HK/2017 virus, and cross-reactivity data generated will be used to better inform the choice of antigen. More studies are certainly needed and may include vaccines manufactured using multiple platforms, e.g., recombinant, cell-based, and/or egg-based antigens.

Taken together, recombinant H7 was safe and well-tolerated, with AEs reported being expected reactions to influenza vaccination, and the safety and immunogenicity of the recombinant H7 is comparable to that of the H7N9 vaccines produced in eggs or cells in response to the emergence of these viruses in 2013. While the NPIVS has an important role in a pandemic influenza preparedness and response, it is not the sole solution due to the ever-present antigenic evolution of influenza viruses. Therefore, the US must continue to develop more effective seasonal influenza vaccines and maintain sustainable domestic manufacturing capacity to rapidly produce, release, and deliver several hundred million doses of strain-matched influenza virus vaccines and dose-sparing adjuvant during a pandemic response. In addition, all efforts must continue to develop a universal influenza vaccine with broad and long-lasting immunity to protect from seasonal and pandemic influenza viruses.

## Methods

### Study design

This was a double-blind, randomized, Phase 2 clinical study (Clinical Trials.gov identifier: NCT03283319; registration date: September 12, 2017) that assessed the safety and immunogenicity of two doses of monovalent influenza recombinant H7 vaccine at three antigen dose levels (3.75, 7.5, and 15 µg) adjuvanted with AS03 or MF59 administered 28 days apart. This study was conducted in healthy male and non-pregnant female adults aged 18–49 years. It was conducted at four clinical research sites in the US between October 2017 and November 2018 in accordance with Good Clinical Practice guidelines, Declaration of Helsinki, and all applicable regulations. All study-related documents were approved by FDA, BARDA, and an institutional review board. Written informed consent was obtained from all enrolled subjects.

### Study vaccine

The vaccine used in this study was recombinant H7, a monovalent H7N9 recombinant HA derived from A/Guangdong/17SF003/2016 (H7N9) HPAI virus, manufactured under a licensed process in the baculovirus expression vector system by Protein Sciences Corporation (Meriden, CT)^31^. AS03 adjuvant (GlaxoSmithKline, GSK) and MF59 adjuvant (Seqirus) were provided by the BARDA-managed NPIVS. Vaccines and adjuvants used in this study passed all release tests and met all specifications for both drug substance and final formulated drug product, and data were submitted, reviewed, and are on file as part of the Investigational New Drug (IND) application to FDA for this study. Subjects were randomized to receive one of three antigen dose levels (3.75, 7.5, or 15 μg) mixed 1:1 with adjuvant at the time of vaccination and administered intramuscularly (IM) as a 0.5 mL dose.

### Safety assessment

The primary safety endpoints were solicited local or systemic reactogenicity symptoms that occurred within 8 days of each vaccine administration. These reactogenicity symptoms consisted of the following: solicited local reactions at the injection site (erythema/redness, induration/swelling, and pain) and solicited systemic reactions (fever, myalgia, arthralgia, fatigue, headache, nausea, vomiting, diarrhea, and chills). The AEs grading scale followed guidelines established by the Division of AIDS, NIH (i.e., mild, Grade 1; moderate, Grade 2; severe, Grades 3, 4, and 5)^32^.

Secondary safety assessments included unsolicited AEs from the time of the first vaccination through 21 days after the second vaccination. Additionally, venous blood samples for routine clinical laboratory safety evaluations were obtained at screening and 8 days after each of the two vaccinations. Secondary safety assessments included serious adverse events (SAEs), medically attended adverse events (MAAEs), and PIMMCs through 13 months following the first vaccine administration.

### Immunogenicity assessment

Serum was collected prior to vaccination on day 1 and on days 29 (prior to second dose), 50, 121, and 212. HAI and MN assays were performed by Southern Research (Birmingham, AL) using Good Laboratory Practice as previously reported^16^ against antigenic variants including reassortant homologous A/Guangdong/17SF003/2016xPR8 (CBER-RG7C, H7N9, FDA, Silver Spring, MD) or heterologous A/Hong Kong/125/2017xPR8 (IDCDC-RG56B, H7N9, CDC, Atlanta, GA), and A/Shanghai/02/2013xPR8 (IDCDC-RG32A, H7N9, CDC). The HAI and MN assays were qualified prior to immunogenicity assessment to determine the limit of detection (LOD) and to define the lowest (lower limit of quantitation, LLOQ) and highest (upper limit of quantitation, ULOQ) amount of analyte that could be measured with acceptable precision and accuracy (Table 3). Together, the ULOQ and LLOQ define the range of quantification for the respective assays, and results outside these ranges may be uncertain. Positive controls in the serological assays included ferret antisera to A/Guangdong/17SF003/2016xPR8 (St. Jude Children’s Research Hospital, Memphis, TN; Lot # G.21021), A/Hong Kong/125/2017xPR8 (St. Jude Children’s Research Hospital; Lot # 633551), and A/Shanghai/02/2013xPR8 (Lot # 61968307) (International Reagent Resource, Manassas, VA). Human serum (Aldrich-Sigma, St. Louis, MO) was included as a negative control in the assays. Briefly, sera were adsorbed with horse red blood cells (HRBCs, Lampire Biological Laboratories, Pipersville, PA) and tested for non-specific agglutination. Agglutinated or partially agglutinated samples underwent RBC adsorption a second time. HRBC-adsorbed serum samples were treated with receptor-destroying enzyme (RDEII, Denka-Seiken, Tokyo, Japan) to inactivate non-specific serum inhibitors. Serial, 2-fold dilutions of the HRBC-adsorbed and heat-inactivated serum were incubated with 4 hemagglutination units of the appropriate influenza virus to allow antigen–antibody binding. An equal volume of 0.5% HRBCs was added to each well. HAI titers were determined as the reciprocal of the highest serum dilution that completely inhibited hemagglutination. Sera tested for MN activity were heat-inactivated to remove non-specific inhibitors. Serial, 2-fold dilutions of inactivated serum were incubated with 100 TCID_50_/50 µl to allow for antigen–antibody binding. MDCK cells (1.5 × 10^4^ cells; London cell line; Sigma-Aldrich, St. Louis, MO) were added to each well and incubated overnight. Cells were fixed in 80% acetone-PBS. ELISAs were performed to determine HA-specific IgG endpoint titers, and MN titers were defined as the reciprocal of the highest dilution of serum that gave 50% neutralization.

### Statistical analysis

SPRs were defined as the proportion of subjects achieving a serum HAI antibody titer ≥40 against homologous antigen, and the 95% exact confidence intervals were determined for each individual group. GMTs and back-transformed 95% CIs based on the *t* distribution were summarized. The reverse cumulative distribution of GMTs were also plotted. In addition, antibody titers against different virus strains were presented as a scatterplot with a regression line on the log scale, along with the Pearson correlation coefficient and corresponding *p*-value. For all titer analyses, titer values marked as above the upper LOD were imputed as the upper LOD and titer values marked as below the lower LOD were imputed for titer analyses as half of the lower LOD.

### Reporting summary

Further information on research design is available in the Nature Research Reporting Summary linked to this article.

## Supplementary information

Reporting Summary

Supplementary Information

## Data Availability

The data generated and analyzed during the current study are available from the corresponding author on reasonable request.
