# Supplementary material for: Adjuvanted recombinant hemagglutinin H7 vaccine to highly pathogenic influenza A(H7N9) elicits high and sustained antibody responses in healthy adults
Source: NPJ Vaccines. 2021 Mar 19;6:41. doi: 10.1038/s41541-021-00287-7 (PMC7979905; doi:10.1038/s41541-021-00287-7)
Supplement: Supplementary file 1 — Reporting Summary [file 41541_2021_287_MOESM1_ESM.pdf]

nature research

Corresponding author(s): Ruben Donis

Last updated by author(s): Dec 18, 2020

Reporting Summary

Nature Research wishes to improve the reproducibility of the work that we publish. This form provides structure for consistency and transparency in reporting. For further information on Nature Research policies, see our Editorial Policies and the Editorial Policy Checklist.

Statistics

For all statistical analyses, confirm that the following items are present in the figure legend, table legend, main text, or Methods section.

n/a	Confirmed	
☐	☒	The exact sample size (n) for each experimental group/condition, given as a discrete number and unit of measurement
☐	☒	A statement on whether measurements were taken from distinct samples or whether the same sample was measured repeatedly
☐	☒	The statistical test(s) used AND whether they are one- or two-sided<br>*Only common tests should be described solely by name; describe more complex techniques in the Methods section.*
☐	☒	A description of all covariates tested
☒	☐	A description of any assumptions or corrections, such as tests of normality and adjustment for multiple comparisons
☐	☒	A full description of the statistical parameters including central tendency (e.g. means) or other basic estimates (e.g. regression coefficient) AND variation (e.g. standard deviation) or associated estimates of uncertainty (e.g. confidence intervals)
☐	☒	For null hypothesis testing, the test statistic (e.g. F, t, r) with confidence intervals, effect sizes, degrees of freedom and P value noted<br>*Give P values as exact values whenever suitable.*
☒	☐	For Bayesian analysis, information on the choice of priors and Markov chain Monte Carlo settings
☒	☐	For hierarchical and complex designs, identification of the appropriate level for tests and full reporting of outcomes
☐	☒	Estimates of effect sizes (e.g. Cohen's d, Pearson's r), indicating how they were calculated

Our web collection on statistics for biologists contains articles on many of the points above.

Software and code

Policy information about availability of computer code

Data collection	No software was used
Data analysis	Statistical analyses was performed using SAS® software Version 9.3 or later.

For manuscripts utilizing custom algorithms or software that are central to the research but not yet described in published literature, software must be made available to editors and reviewers. We strongly encourage code deposition in a community repository (e.g. GitHub). See the Nature Research guidelines for submitting code & software for further information.

Data

Policy information about availability of data

All manuscripts must include a data availability statement. This statement should provide the following information, where applicable:

- Accession codes, unique identifiers, or web links for publicly available datasets
- A list of figures that have associated raw data
- A description of any restrictions on data availability

The data generated and analyzed during the current study are available from the corresponding author on reasonable request.

Field-specific reporting

Please select the one below that is the best fit for your research. If you are not sure, read the appropriate sections before making your selection.

☒ Life sciences ☐ Behavioural & social sciences ☐ Ecological, evolutionary & environmental sciences

For a reference copy of the document with all sections, see nature.com/documents/nr-reporting-summary-flat.pdf

Life sciences study design

All studies must disclose on these points even when the disclosure is negative.

Sample size	No formal power analyses were conducted, since the study objectives require no hypothesis testing. The sample size for this study is approximately 360 subjects, randomized 1:1:1:1:1:1into 6 treatment groups of approximately 60 subjects each. This sample size is consistent with previous studies involving influenza vaccination.
Data exclusions	None
Replication	This study was a clinical trial based on subjects being randomized to a study treatment.
Randomization	Subjects were randomized to study treatment only if they met all of the inclusion criteria and none of the exclusion criteria. Subjects were randomly assigned to 1 of 6 treatment groups in a 1:1:1:1:1:1 ratio. An interactive web response system (IWRS) was used to centrally administer the randomization schedule. The randomization schedule was generated using SAS software Version 9.3 or later (SAS Institute Inc., Cary, North Carolina). Randomization occurred according to a fixed schedule using a permuted block design stratified by clinical site. The IWRS assigned subjects to treatment groups based on the predefined randomization list. A kit ID corresponding to the assigned treatment was assigned by the IWRS from the inventory available at the site. The randomization schedule was generated by the unblinded randomization team and was kept strictly confidential, accessible only to authorized unblinded persons.
Blinding	This is a double-blinded study. All study vaccines were prepared and administered by an unblinded study staff member. Study vaccine accountability were monitored by a separate unblinded study monitor. The subject and all other study staff involved in observing the subject after vaccination were blinded to group assignment. Laboratory staff performing the safety laboratory assessments and immunogenicity assays were blinded to treatment group.The study data through Day 212 were unblinded to prepare the clinical study report. The blinded site and laboratory staff were kept blinded at the subject level through the end of study.

Reporting for specific materials, systems and methods

We require information from authors about some types of materials, experimental systems and methods used in many studies. Here, indicate whether each material, system or method listed is relevant to your study. If you are not sure if a list item applies to your research, read the appropriate section before selecting a response.

Materials & experimental systems

n/a	Involved in the study	
☐	☒	Antibodies
☐	☒	Eukaryotic cell lines
☒	☐	Palaeontology and archaeology
☒	☐	Animals and other organisms
☐	☒	Human research participants
☐	☒	Clinical data
☒	☐	Dual use research of concern

Methods

n/a	Involved in the study	
☒	☐	ChIP-seq
☒	☐	Flow cytometry
☒	☐	MRI-based neuroimaging

Antibodies

Antibodies used	See reference 16: Oshansky, C. M. et al. Safety and immunogenicity of influenza A(H5N1) vaccine stored up to twelve years in the National Pre-Pandemic Influenza Vaccine Stockpile (NPIVS). Vaccine 37, 435-443, doi:10.1016/j.vaccine.2018.11.069 (2019).
Validation	The HAI and MN assays were qualified prior to immunogenicity assessment to determine the limit of detection (LOD) and to define the lowest (lower limit of quantitation; LLOQ) and highest (upper limit of quantitation; ULOQ) amount of analyte that could be measured with acceptable precision and accuracy.

Eukaryotic cell lines

Policy information about cell lines

Cell line source(s)	See reference 16: Oshansky, C. M. et al. Safety and immunogenicity of influenza A(H5N1) vaccine stored up to twelve years in

Cell line source(s)	the National Pre-Pandemic Influenza Vaccine Stockpile (NPIVS). Vaccine 37, 435-443, doi:10.1016/j.vaccine.2018.11.069 (2019).
Authentication	See reference 16: Oshansky, C. M. et al. Safety and immunogenicity of influenza A(H5N1) vaccine stored up to twelve years in the National Pre-Pandemic Influenza Vaccine Stockpile (NPIVS). Vaccine 37, 435-443, doi:10.1016/j.vaccine.2018.11.069 (2019).
Mycoplasma contamination	See reference 16: Oshansky, C. M. et al. Safety and immunogenicity of influenza A(H5N1) vaccine stored up to twelve years in the National Pre-Pandemic Influenza Vaccine Stockpile (NPIVS). Vaccine 37, 435-443, doi:10.1016/j.vaccine.2018.11.069 (2019).
Commonly misidentified lines (See ICLAC register)	See reference 16: Oshansky, C. M. et al. Safety and immunogenicity of influenza A(H5N1) vaccine stored up to twelve years in the National Pre-Pandemic Influenza Vaccine Stockpile (NPIVS). Vaccine 37, 435-443, doi:10.1016/j.vaccine.2018.11.069 (2019).

Human research participants

Policy information about studies involving human research participants

Population characteristics	Healthy males and nonpregnant females aged 18 to 49 years.
Recruitment	Subjects were recruited by the individual investigators by using IRB approved advertisements and from their existing subject databases. Subjects were randomized to 1 of 6 treatment groups in a 1:1:1:1:1:1 ratio and received study treatment only if they met all of the inclusion criteria and none of the exclusion criteria. In addition, in order to receive the second vaccination, subjects must have had all of the inclusion and exclusion criteria assessed; if the subject no longer met eligibility criteria, the investigator, in consultation with the medical monitor in cases of uncertainty, was to determine whether the subject should receive the second vaccination or be terminated early from study vaccination. Subjects who did not receive the second vaccination were to be followed as defined in the Study Protocol.
Ethics oversight	BARDA, Rho, Inc., and the institutional review board

Note that full information on the approval of the study protocol must also be provided in the manuscript.

Clinical data

Policy information about clinical studies

All manuscripts should comply with the ICMJE guidelines for publication of clinical research and a completed CONSORT checklist must be included with all submissions.

Clinical trial registration	NCT03283319
Study protocol	The full study protocol can be accessed at https://clinicaltrials.gov/ct2/show/NCT03283319 under "Study Documents."
Data collection	The study was conducted at four clinical research sites in the US between October 2017 and November 2018.
Outcomes	Study objectives were predefined in the study protocol as below. Further the full statistical analysis plan can be accessed at https://clinicaltrials.gov/ct2/show/NCT03283319 under "Study Documents."<br>Primary Safety Objective:<br>• To assess the safety and reactogenicity for 8 days postvaccination, inclusive of the vaccination day (Day 1 through Day 8 and Day 29 through Day 36), of 3 different antigen dosages of Panblok H7 vaccine given with AS03 or MF59 adjuvant (henceforth referred to as “study vaccines”) administered intramuscularly (IM) on Days 1 and 29, as determined by solicited local and systemic reactogenicity symptoms.<br>Primary Immunogenicity Objective:<br>• To assess the serum hemagglutination-inhibition (HAI) antibody seroprotection rate on Day 50 of 3 different antigen dosages of study vaccines administered IM on Days 1 and 29.<br>Secondary Safety Objective<br>• To assess the occurrence of unsolicited adverse events (AEs), serious adverse events (SAEs), and medically attended adverse events (MAAEs) including a subset of specific potentially immune-mediated medical conditions (PIMMCs) in the 6 treatment groups for 13 months after the first dose of study vaccine.<br>Secondary Immunogenicity Objectives<br>• To assess the serum HAI antibody titers, seroprotection rates, and seroconversion rates of 3 different antigen dosages of the study vaccines through Day 212.<br>• To assess the serum microneutralization (MN) antibody titers and seroconversion rates of 3 different antigen dosages of the study vaccines through Day 212.
