# Supplementary material for: Adjuvanted recombinant hemagglutinin H7 vaccine to highly pathogenic influenza A(H7N9) elicits high and sustained antibody responses in healthy adults
Source: NPJ Vaccines. 2021 Mar 19;6:41. doi: 10.1038/s41541-021-00287-7 (PMC7979905; doi:10.1038/s41541-021-00287-7)
Supplement: Supplementary file 2 — Supplementary Information [file 41541_2021_287_MOESM2_ESM.pdf]

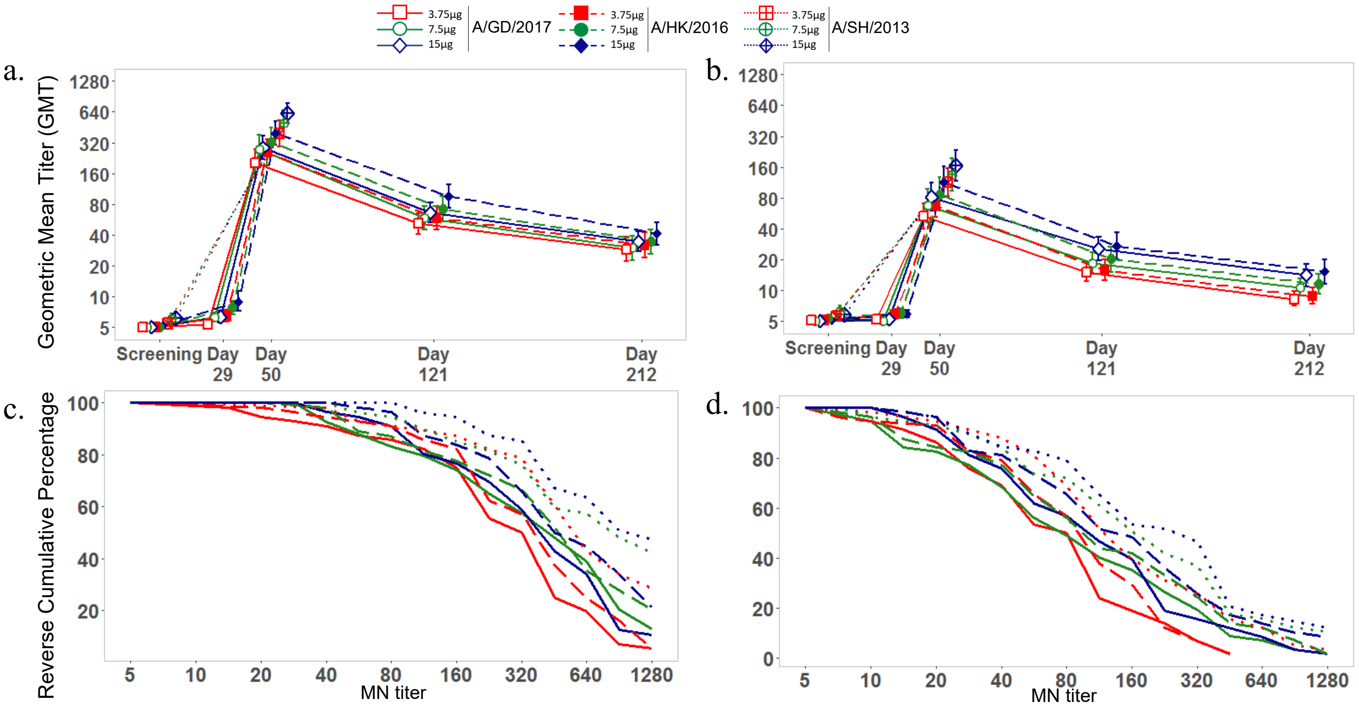

Supplemental Figure 1. Adjuvanted recombinant H7 at 3.75ug, 7.5ug, and 15ug doses elicits neutralizing antibody responses in all study groups. Microneutralization (MN) antibody responses (a, b), and reverse cumulative % MN responses (c, d) to homologous A/Guangdong/17SF003/2016 (H7N9) and heterologous A/Hong Kong/125/2017 (H7N9) fifth epidemic viruses as well as heterologous A/Shanghai/02/2013 (H7N9) first epidemic virus (day 1 and 50 only). Antibody results to AS03-adjuvanted recombinant H7 (left) and MF59-adjuvanted recombinant H7 (right) are shown as geometric mean titer (GMT); 95% confidence interval (CI; vertical bars).
